# Exosomes in corneal diseases: advances in diagnosis and therapy

**DOI:** 10.3389/fcell.2026.1837531

**Published:** 2026-06-30

**Authors:** Xuan Liao, Hongkai Gao, Mengtian Bai, Wei Chi, Weihua Yang

**Affiliations:** 1 Department of Ophthalmology, Affiliated Hospital of North Sichuan Medical College, Nanchong, China; 2 Medical School of Ophthalmology and Optometry, North Sichuan Medical College, Nanchong, China; 3 Shenzhen Eye Hospital, Shenzhen Eye Medical Center, Southern Medical University, Shenzhen, China

**Keywords:** biomarker, corneal disease, engineered exosomes, exosome, extracellular vesicle, isolation standardization, regenerative therapy

## Abstract

The diagnosis and treatment of corneal diseases still face many challenges. As nanoscale vesicles (30–150 nm in diameter), exosomes facilitate intercellular communication by transporting bioactive molecules such as proteins and nucleic acids. Exhibiting low immunogenicity, minimal toxicity, and engineering versatility, they hold strong promise for both diagnostic and therapeutic applications in corneal diseases. This review provides a comprehensive overview of the current understanding and future directions of exosome research in corneal diseases. We systematically compare traditional and emerging methods for exosome isolation and purification. We then discuss their expanding roles: (1) as diagnostic biomarkers and (2) as therapeutic agents or drug delivery systems, with an emphasis on representative examples and engineering innovations. Finally, we critically assess persistent challenges and outline essential research priorities for clinical translation. Looking forward, we highlight the need for standardized isolation protocols, large-scale production of clinical-grade exosomes, and targeted engineering strategies (*e.g.*, surface modification or cargo loading) to enhance corneal wound healing and immune modulation. The integration of exosome-based therapies with biomaterials and sustained-release platforms may further unlock their potential, paving the way for personalized, off-the-shelf treatments for corneal disorders.

## 1 Introduction

Acting as the central refractive component of the eye, the cornea connects to the sclera, with transparency ensured by its distinct layers: the epithelium, Bowman’s layer, stroma, Descemet’s membrane, and endothelium. Direct environmental exposure increases vulnerability to physicochemical trauma, potentially inducing inflammation, ulceration, or scarring that may cause vision loss when improperly managed. The diagnosis and treatment of corneal diseases still face many challenges. Common corneal pathologies include infectious keratitis, keratoconus (KC), dry eye disease (DED), corneal neovascularization, chemical burns, and endothelial dystrophies (Ong Tone et al., 2021; Roshandel et al., 2018; Santodomingo-Rubido et al., 2022; Messmer, 2015; Cabrera-Aguas et al., 2022). Corneal pathologies are the second leading cause of blindness globally, and corneal trauma accounts for a majority of eye-related emergency room visits (Yeo et al., 2026; Xie et al., 2026). The demand for donor corneas vastly exceeds supply—nearly 13 million patients worldwide are currently awaiting transplantation (Gain et al., 2016). Conventional treatments rely on antibiotics, anti-inflammatory agents, amniotic membrane transplantation, or corneal grafting. However, these approaches face substantial drawbacks: antimicrobial resistance continues to escalate, with multidrug-resistant strains of *Pseudomonas aeruginosa* and *Staphylococcus aureus* increasingly implicated in treatment-refractory infectious keratitis; steroid-related complications including cataract formation, elevated intraocular pressure, and corneal melting for medical therapy; donor tissue scarcity and immune rejection for keratoplasty, with 5-year graft survival rates as low as 50%–70% in high-risk recipients; as well as high recurrence rates and limited efficacy in advanced or refractory cases (Tan et al., 2012). Corneal healing critically depends on efficient re-epithelialization. Limited regenerative capacity in specific layers such as the endothelium and persistent inflammatory responses often lead to suboptimal repair accompanied by stromal haze and fibrosis. Given these clinical challenges, novel therapeutic strategies that can modulate inflammation, promote regeneration, and bypass immune rejection are urgently needed.

Emerging evidence reveals that exosomes—nanoscale extracellular vesicles naturally involved in intercellular communication—actively participate in corneal repair, spurring growing interest in their use as natural nanocarriers for diagnostic and targeted therapies (Jeon et al., 2025; Pedersen et al., 2024; Guo et al., 2026). The importance of exosomes lies in their ability to transfer bioactive cargos (proteins, lipids, and nucleic acids) that recapitulate the therapeutic effects of parent cells while avoiding direct cell transplantation. Unlike conventional pharmacological agents that typically target a single pathway, exosomes simultaneously regulate multiple pathological processes—including inflammation, oxidative stress, and fibrosis—through their diverse cargo repertoire. Compared with cell-based therapies, exosomes offer comparable regenerative and immunomodulatory efficacy while circumventing the risks of immune rejection, tumorigenicity, and the logistical complexities of cold-chain cell storage. Furthermore, their lipid bilayer architecture confers superior stability in biofluids relative to recombinant growth factors, whose short half-lives necessitate frequent administration. These properties make them particularly attractive for addressing the complex microenvironment of corneal diseases, where single-target therapies often fail.

Exosomes form a distinct subclass of EVs, defined by their special morphology and diameters ranging from 30 to 150 nm. Exosome formation is initiated through the endosomal pathway where multivesicular bodies (MVBs) develop via inward membrane budding to produce intraluminal vesicles. The MVBs fuse with the plasma membrane, releasing these internal vesicles as exosomes into the extracellular space (Krylova and Feng, 2023). Exosomes are present widely in biofluids such as blood and aqueous humor, transporting nucleic acids, proteins, and lipids that reflect their parent cells. They facilitate intercellular communication through signal transduction and molecular transfer to regulate anti-inflammatory responses and tissue regeneration. These inherent traits—low immunogenicity, minimal toxicity, and engineerability—make exosomes strong candidates for biomedical applications. Despite their potential for corneal diseases, including dry eye, corneal injury, and neovascularization, natural exosomes face significant research hurdles before clinical translation. One of the most severe limitations is that natural exosomes possess poor targeting specificity and low drug-loading capacity, alongside challenges in scalable, standardized production. Moreover, conventional single-modification approaches remain insufficient for addressing the complex pathological microenvironment of corneal injuries, which involves interconnected inflammatory, fibrotic, and oxidative stress processes. This review systematically compares nine isolation and purification methodologies, presents disease-specific molecular signatures for major corneal pathologies, and critically examines the translational barriers—including delivery hurdles, toxicity considerations, regulatory frameworks, and manufacturing scalability—that must be overcome to enable clinical adoption. By clarifying the intrinsic structure-function-efficacy relationship, this review aims to establish a foundation for developing advanced exosome-based therapeutics and to provide a roadmap for their clinical translation in the management of corneal diseases.

## 2 Isolation and purification

Exosome separation and purification rely on five core techniques: ultracentrifugation, ultrafiltration, precipitation, immunoaffinity capture, and size-exclusion chromatography (SEC) (Massoumi et al., 2023).

Ultracentrifugation remains the most widely used method given its operational simplicity and high yield. Density-gradient ultracentrifugation using sucrose or iodixanol gradients can further improve EV purity by reducing co-isolated protein aggregates and lipoproteins, though at the cost of additional processing time. However, its long processing time (approximately 2 h, as per the typical protocol: 10 min at 300 g → 10 min at 2,000 g → 30 min at 10,000 g → 70 min at 100,000 g, all at 4 °C) and moderate purity levels limit its utility for proteomic analyses requiring high-purity samples (Li et al., 2017; Livshits et al., 2015). Additionally, high-speed centrifugation can induce vesicle aggregation and membrane damage, while co-sedimentation of lipoproteins and protein aggregates compromises purity (Choi et al., 2026; Lundy et al., 2026).

Ultrafiltration enables rapid sample processing by using membranes of defined pore sizes to isolate and concentrate exosomes typically larger than 30 nm. This rapid method suffers from shear-induced damage to exosome integrity and membrane fouling, substantially reducing yield (Lobb et al., 2015).

Polymer-based precipitation using polyethylene glycol (PEG) offers a cost-effective and straightforward approach. Common protocols use polyethylene glycol with molecular weights from 6,000 Da to 20,000 Da, often polyethylene glycol 6,000, at an 8% (w/v) concentration and a working range of 5%–20% depending on the sample type. The procedure involves overnight incubation of the sample-PEG mixture at 4 °C with 1 M NaCl to enhance exosome recovery. Unfortunately, the purity of the exosomes is relatively low (Li et al., 2017; Huang et al., 2025).

Immunoaffinity capture employs antibodies directed against exosomal surface markers such as CD63, CD9, and CD81 to isolate highly pure exosome populations while preserving their biological activity. Nevertheless, the high reagent costs associated with this method limit its scalability (Chen J. et al., 2021). Moreover, this approach selectively enriches specific EV subpopulations, potentially excluding therapeutically relevant vesicles lacking the targeted marker (Choi et al., 2026).

SEC is a chromatography technique that separates exosomes by hydrodynamic radius by pumping the sample through a perforated gel-packed column (*e.g.*, agarose or dextran). Larger particles unable to enter the gel pores elute first, followed by medium-sized particles like exosomes in subsequent fractions. Maintaining a low flow rate is essential for high-purity separation by ensuring sufficient resolution between particle populations. This approach ensures effective separation of exosomes from contaminants, yielding structurally intact, high-purity exosomes under mild, shear-free conditions; its high consumable costs and low throughput hinder widespread adoption (Li et al., 2017; Sidhom et al., 2020). Tangential flow filtration (TFF), a gentler variant employing cross-flow dynamics, minimizes membrane fouling and achieves >95% recovery, making it suitable for processing large-volume cell culture supernatants (Kimiz-Gebologlu and Oncel, 2025). When coupled with SEC as a subsequent polishing step, TFF–SEC workflows yield particle-to-protein ratios approaching 1 × 10^9^ particles/μg protein, substantially reducing albumin contamination (Lundy et al., 2026). For ophthalmological applications, isolating EVs from low-volume ocular biofluids such as tears and aqueous humor poses additional technical challenges. Tear samples typically yield limited EV quantities, requiring sensitive detection methods or sample pooling. Microfluidic platforms and single-vesicle analysis techniques are particularly promising for processing these volume-limited clinical specimens, given their low sample consumption and high sensitivity.

Emerging isolation technologies are addressing the purity–yield trade-offs inherent to conventional methods. Asymmetric flow field-flow fractionation (AF4) separates nanoparticles in a thin channel using perpendicular cross-flow, achieving nanometer-scale resolution without shear stress. When combined with density cushion ultracentrifugation, AF4 enables high-purity isolation of blood EVs with minimal lipoprotein contamination, facilitating the identification of novel plasma EV markers such as MYCT1 and TSPAN14 (Lundy et al., 2026). Deterministic lateral displacement (DLD) employs microfluidic pillar arrays to continuously separate EVs by size in a label-free manner, with tunable cutoffs down to 200 nm, though throughput remains limited to microliter-per-minute ranges (Lundy et al., 2026; Schank et al., 2026). The EV-Osmoprocessor (EVOs) leverages osmotic gradients across semi-permeable membranes to achieve 50-fold volumetric reduction and 99.7% albumin removal within 2 h without specialized equipment, offering a simple pre-concentration step prior to SEC. For clinical-scale manufacturing, anion exchange chromatography (AEC) exploits the negative surface charge of EVs to enable rapid, scalable isolation from liter-scale conditioned media with minimal user intervention (Salmond and Williams, 2021).

To achieve standardization across studies, the MISEV2023 guidelines recommend replacing the term “exosome” with “small EVs (sEVs, <200 nm)” unless endosomal origin is experimentally proven (Schank et al., 2026). Furthermore, the common practice of adjusting ultracentrifugation time by K-factor is invalid for fixed-angle rotors; instead, a “cut-off size” approach based on complete sedimentation of a desired vesicle diameter should be employed (Livshits et al., 2015). SEC is considered the most reproducible method for preserving EV integrity, and combination strategies (*e.g.*, tangential flow filtration followed by SEC, or PEG precipitation plus ultracentrifugation) are used by >59% of researchers to balance purity and yield (Lobb et al., 2015; Schank et al., 2026). MISEV2023 further mandates reporting of at least five components: cell source/culture conditions, isolation parameters (g-force, time, cut-off size), particle and protein yields, two positive transmembrane markers (*e.g.*, CD9, CD63, CD81), and one negative marker (*e.g.*, albumin, ApoA1, calnexin) (Schank et al., 2026; Zhang and Fang, 2026). The protein corona adsorbed on EVs varies with isolation method and influences downstream immune recognition, thus requiring documentation.

The principal isolation techniques—spanning ultracentrifugation, ultrafiltration, precipitation, immunoaffinity capture, size-exclusion chromatography, and emerging methods including AF4, DLD, and AEC—differ fundamentally in their underlying principles, operational trade-offs, and suitability for downstream applications. Table 1 provides a systematic comparison of these nine approaches, detailing their mechanisms, relative advantages, critical limitations, and typical use cases to guide method selection based on sample type and experimental objectives.

**TABLE 1:** Comparison of exosomes isolation and purification methods.

Method	Principle	Advantages	Limitations	Typical applications	Ref.
Ultracentrifugation	Sequential sedimentation by size and density under increasing centrifugal force (up to 100,000–200,000×g)	High yield; well-established; low reagent cost; suitable for large sample volumes	Time-consuming (>4 h); rotor-dependent efficiency; moderate purity; potential vesicle damage and aggregation	Initial enrichment from cell culture supernatants and large-volume biofluids; basic research	Li et al. (2017); Livshits et al. (2015); Choi et al. (2026); Lundy et al. (2026)
Ultrafiltration	Size-based retention using membranes with defined pore sizes (*e.g.*, 30 nm, 100 kDa MWCO)	Rapid (<1 h); simple; no specialized equipment; scalable	Membrane fouling and clogging; shear-induced vesicle deformation; loss of EVs due to membrane binding	Concentration of diluted samples; pre-treatment before SEC or density gradient	Lobb et al. (2015)
Polymer-based precipitation	Hydrophilic polymer reduces EV solubility, enabling low-speed precipitation	Simple, fast (overnight incubation); high yield; low cost; scalable	Low purity; co-precipitation of non-EV proteins, lipoproteins, viral particles; residual polymer	Large-scale crude EV enrichment; downstream RNA analysis (if purity is not critical)	Li et al. (2017); Huang et al. (2025))
Immunoaffinity capture	Antibodies (anti-CD9, anti-CD63, anti-CD81, etc.) immobilized on magnetic beads or chips specifically bind EV surface markers	High purity and specificity; isolates EV subpopulations; preserves biological activity	Low yield; high reagent cost; selectively enriches subpopulations; may exclude therapeutically relevant vesicles lacking target marker	Subpopulation-specific EV isolation (*e.g*., tumor-derived EVs); diagnostic applications	Choi et al. (2026); Chen et al. (2021a)
Size-exclusion chromatography (SEC)	Porous gel column (agarose, dextran) separates particles by hydrodynamic radius; larger particles elute first, followed by EVs (30–200 nm)	High purity; preserves vesicle integrity and function; gentle (gravity flow); reproducible	Moderate yield; high column cost; low throughput; requires pre-concentration; cannot separate EVs from similarly sized lipoproteins	Plasma/serum/urine samples; biomarker discovery; functional studies requiring intact EVs	Li et al. (2017); Sidhom et al. (2020)
Asymmetric Flow Field-Flow Fractionation (AF4)	Nanoparticles separated in thin channel by perpendicular cross-flow; diffusion coefficient determines elution order	Nanometer-scale resolution; label-free; gentle (no shear); resolves EV subpopulations	Requires specialized instrumentation; low throughput; needs pre-concentrated samples	High-purity EV isolation from plasma; subpopulation analysis; biomarker discovery	Lundy et al., 2026
Deterministic Lateral Displacement (DLD)	Microfluidic pillar array deflects particles above critical diameter; size-based continuous separation	Label-free; tunable cutoff (down to 200 nm); preserves integrity; continuous flow	Limited throughput (μL/min); channel clogging risk; fabrication complexity	Microfluidic EV sorting; diagnostic sample preparation	Lundy et al. (2026); Schank et al. (2026)
Anion Exchange Chromatography (AEC)	Negatively charged EVs bind to positively charged column; eluted by increasing salt concentration	Rapid; scalable; minimal user intervention; processes liter-scale media within hours	Requires buffer optimization; may co-elute similarly charged contaminants	Large-scale clinical manufacturing; industrial EV production	Salmond and Williams (2021)
Combined strategies (*e.g.*, TFF + SEC, PEG + UC)	Sequential use of two or more methods to balance yield and purity	Higher purity and yield than single method; adaptable to sample type; scalable	More complex and time-consuming; higher overall cost; requires protocol optimization	GMP-grade EV production; clinical sample processing; multi-omics analyses	Li et al. (2017); Lobb et al. (2015); Sidhom et al. (2020); Kimiz-Gebologlu and Oncel (2025)

No single isolation method fulfills all requirements for purity, yield, bioactivity, and throughput across different research applications. Researchers must recognize the advantages and limitations of each isolation technique and employ integrated approaches to balance methodological strengths for optimal downstream application outcomes.

## 3 The potential of exosomes as biomarkers for disease diagnosis

Exosomes package a wide variety of bioactive molecules. This biochemical cargo makeup faithfully mirrors the physiological or pathological state of their parent cells. These characteristics position exosomes as highly valuable targets for disease diagnosis, providing new avenues for the early detection and prevention of corneal diseases (Table 2). Differentially expressed exosome biomarkers hold significant potential for enabling early disease diagnosis and intervention. Table 2 provides a consolidated overview of these disease-associated molecular signatures across major corneal conditions—including keratoconus, DED, primary Sjögren’s syndrome (pSS), neurotrophic keratopathy (NK), diabetic keratopathy (DK), and limbal epithelial disorders—categorized by molecular type (protein, miRNA, circRNA) and annotated with expression changes and implicated pathways. This comparative summary serves as a reference for identifying both shared pathogenic mechanisms and disease-specific candidate biomarkers amenable to clinical translation.

**TABLE 2:** Source-related content differences and biomarker roles.

Disease type	Source	Marker type	Marker name(s)	Expression change	Associated Function/pathway	Ref.
Keratoconus (KC)	Corneal tissue	Protein	PGK1	↑ (KC-specific)	Aberrant energy metabolism	Lozano et al. (2022)
	Corneal tissue	Protein	ACTB, SERPINE1, HGFAC	↑	Catalytic activity, binding, regulatory functions	Lozano et al. (2022)
	Corneal tissue	Protein	HRNR, CTNNB1, DSG1, GSN, LTF, GNAS, SERPINB3	↓	​	Lozano et al. (2022)
	Stromal fibroblasts	Protein	NUAK1, PKLR	↑(4.6-fold, 13.96-fold)	TGF-β1 regulation → glycolytic dysfunction → stromal cell metabolic abnormality	Hadvina et al. (2023)
	Corneal tissue	miRNA	hsa-miR-184, hsa-miR-34a-3p	↑	PAX6 inhibition (ocular development), TNF targeting → pro-inflammation	Lozano et al. (2022)
	Corneal tissue	miRNA	hsa-miR-532-5p	↑(KC-specific)	Negative regulation of apoptosis; protein phosphorylation	Lozano et al. (2022)
	Stromal fibroblasts	miRNA	let-7c-5p, miR-655	↑(3.35-fold, 1.54-fold)	ECM/collagen organization; TGF-β receptor binding	Hadvina et al. (2023)
Dry Eye Disease (DED)	Tear	miRNA	miR-127-5p, miR-139-3p, miR-22-5p	↑	Inflammatory pathways → early-detection biomarker	Pucker et al. (2022)
Primary Sjögren’s Syndrome (pSS)	Tear	Protein	PRDX3, CPNE1	↑	Critical pathway involvement → stable biomarkers	Aqrawi et al. (2017)
	Saliva and tear	cirRNA	circ-IQGAP2, circ-ZC3H6	↑	Highly specific early-diagnostic biomarkers	Chopra et al. (2025)
Neurotrophic Keratopathy (NK)	Tear	Protein	MMP-9, CD171+/CD126+ Exos	↑	Dynamic inflammation monitoring (treatment response)	Pieragostino et al. (2022)
	Tear	Protein	ELANE (week 4), IL6R pathway (week 8)	↑	Inflammatory pathway activation	Pieragostino et al. (2022)
Diabetic Keratopathy (DK)	Plasma	Protein	FLOT2	↓	Insulin response regulation → diagnostic marker	Chen et al. (2021b)
	Limbal stem cells	miRNA	miR-184, miR-200b-3p, miR-200c-3p, miR-103a-3p, miR-107	↑	Insulin signaling/Notch pathways → corneal pathology	Leszczynska et al. (2018)
	Limbal stem cells	miRNA	miR-4516, miR-146a-5p	↓	​	Leszczynska et al. (2018)
	Limbal epithelial cells	miRNA	miR-199a-3p, miR-199b-5p, miR-381-3p	↑	Proliferation, migration, wound healing, key signaling pathways	Verma et al. (2023)
Limbus Epithelial Disorder	Limbal epithelial cells	Protein	ERK1/2 (MAPK1), HSP90A/B, VPS4A/VPS29, ANXA4	↑ (ANXA4 prominent)	Regulation of cell proliferation/differentiation → potential diagnostic targets	Verma et al. (2023)

### 3.1 Exosomal proteins as biomarkers

Proteomic analyses have revealed characteristic protein expression profiles in exosomes derived from patients with corneal diseases. In KC, Lozano et al. (2022) identified 14 differentially expressed proteins in corneal tissue-derived exosomes, including upregulated ACTB, SERPINE1, HGFAC, downregulated HRNR, CTNNB1, DSG1, GSN, LTF, GNAS, SERPINB3, and three proteins (RAPPC6A, AZGP1, CSN2) unique to healthy controls, with PGK1 being KC-specific. Functional analysis indicated involvement in catalytic activity, molecular binding, and regulation. Additionally, Hadvina et al. (2023) reported significant upregulation of NUAK1 (4.6-fold) and PKLR (13.96-fold) in keratoconus stromal fibroblasts, linking these proteins to metabolic abnormalities, proliferation defects, and fibrosis via TGF-β1 and glycolysis pathways, suggesting their diagnostic and therapeutic potential.

In NK, Pieragostino et al. (2022) observed that dynamic changes in the tear exosomes proteome evolved in response to intervention with recombinant human nerve growth factor (rhNGF). Inflammation-related proteins (*e.g.*, ELANE) were significantly upregulated at week 4. By week 8, activation of the IL-6R pathway was associated with an increase in CD171^+^ (neuronal origin) and CD126^+^ (IL-6R carrying) exosomes and was closely linked to CD45^+^ inflammatory exosomes. This clinical study of 8 NK patients demonstrated that tear exosomal MMP-9 and CD171^+^/CD126^+^ exosomes could serve as dynamic biomarkers for monitoring therapeutic response.

In pSS, Aqrawi et al. (27 pSS patients vs. 32 controls) identified stable overexpression of PRDX3 and CPNE1 in tear exosomes, which are involved in TNF-α signaling and B Cell survival, underscoring their diagnostic potential (Aqrawi et al., 2017). Moreover, a systematic review by Chopra *et al.* summarized that upregulated inflammatory proteins (*e.g.*, LCN2, GRN, CALM) in saliva and tear exosomes of pSS patients have promising diagnostic predictability (Chopra et al., 2025). Verma *et al.* characterized limbal epithelial exosomes and identified 2,648 disease-related differentially expressed proteins, noting that exosomal ERK1/2, HSP90 isoforms, VPS4A/VPS29, and upregulated ANXA4 have diagnostic value in diabetes (Verma et al., 2023). Chen KC. et al. (2021) found that FLOT2 (an insulin response regulator) in plasma exosomes from patients with DK was significantly reduced, indicating its potential as a diagnostic biomarker.

Beyond the disease-specific alterations described above, understanding exosome release under normal and stress conditions provides crucial insights into pathological mechanisms. Robciuc *et al.* detected sphingolipid pathway enzymes (ASM, NSM2, neuraminidase) in healthy human tears. Using a human corneal epithelial cell (hCEC) model, they showed that UV-B radiation and hyperosmotic stress induced dose-dependent release of sphingomyelinases, and that stress promoted exosome shedding, revealing a release pathway for these enzymes. This suggests that tear exosomes are valuable non-invasive diagnostic tools for anterior segment diseases (Robciuc et al., 2014).

### 3.2 Exosomal RNA as biomarkers

MicroRNA (miRNA) analysis has revealed characteristics of exosomal RNA that are specific to corneal diseases. In KC, Víctor *et al.* reported aberrant expression of multiple miRNAs, including upregulated hsa-miR-184 (inhibiting PAX6), hsa-miR-34a-3p (involved in inflammatory response), and KC-specific hsa-miR-532-5p, while hsa-miR-3192-5p and hsa-miR-320e were uniquely expressed in healthy controls (Lozano et al., 2022). Additionally, Hadvina *et al.* identified significant upregulation of let-7c-5p (3.35-fold) and miR-655 (1.54-fold) in keratoconus stromal fibroblast-derived exosomes, with functional enrichment in extracellular matrix (ECM) organization, steroid hormone, and TGF-β signaling. A unique subset of miRNAs regulating apoptosis inhibition, protein phosphorylation, and kinase activity (miR-532-5p, -345-5p, -328-3p) was exclusively present in KC exosomes, whereas miR-765 and miR-491-5p were detected only in healthy controls (Hadvina et al., 2023).

In DK, Leszczynska *et al.* detected increased expression of miR-184, -200b-3p, −200c-3p, −103a-3p, and −107, and decreased miR-4516 and -146a-5p in limbal stem cell-derived exosomes, along with alterations in piRNAs, snoRNAs, and Y RNAs, implicating insulin and Notch signaling pathways (Leszczynska et al., 2018). Verma *et al.* found significant alterations in miR-199a-3p, miR-199b-5p, and miR-381-3p in diabetes, which are involved in cell proliferation, migration, and wound repair (Verma et al., 2023).

In DED, a clinical study by Pucker *et al.* (5 DED patients vs. Five controls) detected upregulated expression of inflammation-related miRNAs (miR-127-5p, -139-3p, -22-5p) in tear exosomes, suggesting their potential as early diagnostic biomarkers (Pucker et al., 2022). In pSS, a systematic review by Chopra *et al.* summarized clinical evidence identifying plasma-derived circ-IQGAP2 and circ-ZC3H6 as highly specific early diagnostic biomarkers (Chopra et al., 2025).

## 4 Therapeutic applications of exosomes in corneal diseases

Corneal transplantation remains the primary method for the functional restoration of the cornea; however, donor scarcity and immune rejection frequently lead to undesirable outcomes such as graft failure. Exosomes derived from mesenchymal stem/stromal cells (MSCs) of diverse origins—such as adipose tissue, bone marrow, and umbilical cord—play a crucial role in intercellular communication and tissue repair. Nevertheless, exosomes from non-stem cell sources also exhibit considerable and unique therapeutic potential for treating corneal disorders. Table 3 provides a systematic overview of exosome-based therapeutic strategies across corneal disease models, spanning diverse sources: stem cell-derived (adipose-derived mesenchymal stem cell (ADSC), bone marrow-derived mesenchymal stem cell (BMSC), umbilical cord-derived mesenchymal stem cell (UCMSC), corneal stromal stem cell (CSSC), induced pluripotent stem cell (iPSC)), tissue-derived (hCEC), tumor-derived (B16 melanoma), microbial-derived (*Lactobacillus*), and engineered/synthetic platforms (activated T cell-derived exosome (aT-Exo), light-responsive vesicles). For each source, the table delineates the key active components or engineering strategies, underlying therapeutic mechanisms, and resultant functional effects—ranging from anti-fibrotic and anti-inflammatory actions to accelerated epithelial regeneration and graft survival. This comparative framework illuminates both the mechanistic convergence across diverse exosome sources and the unique functional specializations that inform rational selection for specific corneal pathologies. Microbial-derived and engineered exosome strategies are discussed in detail in Sections 5, 6, respectively.

**TABLE 3:** Summary of exosomes from multiple sources and their roles.

Exosome Source	Disease model	Key active Component/Engineering strategy	Primary therapeutic mechanism	Therapeutic effects	Ref.
Adipose-derived Stem Cells (ADSCs)	Corneal Injury/Scarring	miR-19a	Inhibits HIPK2 → Blocks JNK pathway → Reduces myofibroblast differentiation	Accelerates epithelial migration; Reduces α-SMA; Anti-fibrotic	Ma et al. (2025); Doebele et al. (2010)
Bone Marrow Stem Cells (BMSCs)	Alkali Burn/Transplant Rejection	miR-181d-5p (Rapamycin-primed Exosomes - Rapa-Exo)	Targets KLF6 → Inhibits pro-inflammatory cytokines/CCR7	Prolongs graft survival; Inhibits neovascularization; Reduces edema	Xie and Jie (2025); Wei et al. (2023)
Umbilical Cord MSCs (UCMSCs)	Dry Eye Disease (DED)	miR-125b-5p, let-7b-5p	Inhibits IRAK1/TAB2/NF-κB pathway	Alleviates ocular surface inflammation; Improves tear secretion	Wang et al. (2023b)
Corneal Stromal Stem Cells (CSSCs)	Corneal Scarring	miR-29a, miR-381-5p	Targets collagen synthesis/NF-κB signaling → Downregulates α-SMA, Col3A1	Inhibits fibrosis; Maintains corneal transparency	(Yam et al., 2023; Wang et al., 2023a)
Induced Pluripotent Stem Cells (iPSCs)	Corneal Epithelial Defect	Unspecified (Native Exosomes)	Upregulates stem cell markers (P63/PAX6)	Superior repair efficacy to MSC-Exos; Accelerates epithelial regeneration	Wang et al. (2020); Lee et al. (2024a)
Human Corneal Epithelial Cells (hCECs)	Wound Healing	Thrombospondin-1, LTBP-1, CCL2	Activates STAT3/p38α/β-catenin pathway → Promotes migration/proliferation	Accelerates wound closure; Note: High Thrombospondin-1 may promote scarring	Li et al. (2025); Desjardins et al. (2022)
Melanoma Cells (B16-Exo)	Corneal Transplant Rejection	JAK2 protein	Enhances MDSC activity → Suppresses T-cell proliferation (via JAK-STAT pathway)	Inhibits neovascularization; Improves graft survival	Yang et al. (2025)
Engineered Exosomes (aT-Exo)	Dry Eye Disease/Inflammation	Surface-conjugated Anti-TNF-α antibody	Synergistically enhances anti-inflammatory and reparative functions	Improved targeted delivery efficiency; Significantly reduces inflammation	Yu et al. (2024)
*Lactobacillus*	Conjunctivitis	Unspecified (Native Exosomes)	Downregulates NFAT5/NF-κB1 (33%–36%) → Inhibits IL-20/IL-8, *etc.* (Internalized via gut-eye axis)	Alleviates conjunctival inflammation (non-toxic)	Lee et al. (2024b)
Light-responsive Polymer Vesicles	Corneal Injury Summary of exosomes from multiple sources and their roles	NO donors (oNBN/pNBN)	Light-triggered NO release → Promotes epithelial migration/proliferation	Accelerates wound healing; No cytotoxicity	Duan et al. (2020)

### 4.1 Combat corneal scar formation

Corneal injury triggers keratocyte transformation into human corneal fibroblasts (hCFs), which differentiate into human corneal myofibroblasts (HCMs) under TGF-β1 stimulation. HCMs express α-smooth muscle actin (α-SMA) and remodel the ECM by packaging MMPs, CXCL6, and CXCL12 into exosomes; their persistence leads to aberrant wound healing and fibrosis (Yeung et al., 2022). Stem cell-derived exosomes alleviate corneal scars and demonstrate therapeutic efficacy in limbal stem cell deficiency, dry eye-associated epithelial damage, and keratoconus (Kobal et al., 2024).

Adipose-derived mesenchymal stem cell exosomes (ADSC-Exos) enhance epithelial migration, suppress apoptosis via Bax/Bcl-2, and reduce α-SMA expression. In a rat model of corneal alkali injury, topical instillation of ADSC-Exos significantly accelerated re-epithelialization, achieving complete wound closure by day 3 post-injury compared to day 7 in PBS-treated controls. Notably, ADSC-Exos treatment markedly reduced the expression of α-smooth muscle actin (α-SMA) in the corneal stroma and downregulated the pro-apoptotic protein Bax while upregulating the anti-apoptotic protein Bcl-2, thereby concurrently attenuating stromal fibrosis and apoptosis (Ma et al., 2025). Although ADSC-Exos promote fibroblast differentiation, they concurrently reduce MMP and ECM deposition (Shen et al., 2020). This paradox is explained by miR-19a delivery, which inhibits HIPK2 and the JNK-driven fibrotic cascade, guiding cells toward a healthier repair phenotype (Doebele et al., 2010).

Other stem cell-derived exosomes act similarly. In a mouse phototherapeutic keratectomy model, MSC-Exos downregulate Col3a1 and α-SMA via miRNA transfer, preserving corneal transparency (Ong et al., 2023). Bone marrow MSC-Exos (BMSC-Exos) activate p44/42 MAPK (ERK) signaling to enhance epithelial migration, and attenuate inflammation and α-SMA expression *in vivo* (Zhou et al., 2023). In severe alkaline burn models, bone marrow- and umbilical cord-derived exosomes downregulate TNF-α, IL-1β, IL-8, NF-κB, inhibit caspase-8, and suppress pathological angiogenesis (Tao et al., 2019; Saccu et al., 2022).

Furthermore, corneal stromal stem cell exosomes are enriched with miR-29a and miR-381-5p. These miRNAs target pathways involved in collagen synthesis, NF-κB signaling, and the expression of fibrosis markers such as α-SMA and Col3A1. The expression levels of these miRNAs can be increased more than 20-fold, demonstrating potent anti-fibrotic activity (Yam et al., 2023; Wang et al., 2023a). Other miRNAs, including miR-663b, miR-16-5p, and miR-1290, contribute to repair by downregulating the Notch signaling pathway in corneal epithelial stem cells (Yam et al., 2023).

Therapeutic efficacy depends on exosome source; MSC-Exos outperform serum-derived exosomes due to enriched bioactive cargo. Engineering strategies further optimize potential. For example, exosomes from melatonin-pretreated human corneal limbal MSCs efficiently deliver miR-29, drastically reducing TGF-β1 (∼1000-fold) and α-SMA while upregulating TGF-β3 (∼1.6-fold) and PPAR-γ (Altug et al., 2024).

In summary, stem cell-derived exosomes synergistically accelerate corneal cell migration and proliferation, regulate ECM balance, inhibit myofibroblast differentiation and inflammation, and mitigate scar formation. Their efficacy is intrinsically determined by cellular origin: human iPSC-derived exosomes show superior corneal epithelial repair compared to MSC-Exos (Wang et al., 2020; Lee S. et al., 2024), and BMSC-Exos exhibit better wound closure, anti-apoptosis, and immunomodulation than exosomes from corneal epithelial cells (Tati et al., 2024).

### 4.2 Treatment of corneal ulcers and alkali burns

Corneal ulcers commonly result from severe inflammatory reactions and can lead to significant visual impairment. Meissner et al. (2024) identified equine adipose-derived mesenchymal stem cell exosomes (eqADSC-Exos) as a therapeutic agent for corneal injury. Their mechanisms include: enhancing mitotic activity and cell proliferation; inhibiting pro-inflammatory cytokines to alleviate inflammation; and upregulating miR-146a-5p, a key anti-inflammatory molecule. Furthermore, the study revealed that eqADSC-Exos treatment increases mitochondrial content and reduces Fis-1/Parkin levels, thereby maintaining the homeostasis of corneal stromal cells and inhibiting their apoptosis. This mechanism reinforces the therapeutic effect by targeting mitochondrial function. These equine-derived findings offer valuable comparative insights; however, differences in corneal anatomy, wound healing dynamics, and immune responses between horses and humans warrant species-specific validation when extrapolating to human corneal disease.

While the equine ADSC-Exos study provides valuable mechanistic insights, preclinical validation in large-animal models of corneal alkali burns further underscores the translational potential of MSC-derived exosomes. In a rabbit model of severe alkali burn, subconjunctival injection of BMSC-Exos significantly reduced corneal opacity scores and suppressed corneal neovascularization by approximately 65% compared to PBS controls within 14 days. Importantly, the therapeutic efficacy of BMSC-Exos was comparable to that of topical corticosteroid treatment but without the associated risk of elevated intraocular pressure, highlighting the safety advantage of exosome-based interventions (Xie and Jie, 2025). While these findings remain at the preclinical stage, they represent relatively high-level translational evidence currently available for exosome-based therapies in corneal alkali burns and provide a compelling rationale for future clinical development.

### 4.3 Intervention for corneal endothelial malnutrition

A major cause of blindness is corneal endothelial dysfunction, with its pathogenesis intimately linked to endoplasmic reticulum stress (ER stress). Buono et al. (2021) investigated the protective mechanisms of MSC-Exos on hCECs under ER stress, revealing multi-level regulatory pathways: (1) MSC-Exos counteract ER stress-induced apoptosis via a dual mechanism: they attenuate eIF2α phosphorylation while concurrently activating the pro-survival Akt pathway, collectively enhancing cell viability; (2) Exertion of anti-apoptotic effects: MSC-Exos significantly reduce caspase-3 activity and inhibit the apoptotic process; (3) Functional miRNA transfer: MSC-Exos deliver highly expressed miRNAs (hsa-miR-222-3p, hsa-miR-125b-5p, and hsa-miR-21-5p) to hCECs. By delivering miRNAs that target key ER stress molecules, MSC-Exos regulate gene expression post-transcriptionally, thereby further enhancing cellular stress adaptation. This study elucidates the protective role of MSC-Exos in the corneal endothelium and establishes a molecular foundation for future clinical application.

### 4.4 Inhibiting transplant rejection and promoting vascular reconstruction

The primary causes of corneal transplant failure are immune rejection and limbal vascular reconstruction disorders. Engineered exosomes have shown advantages in suppressing immune rejection. The team led by Chao Wei et al. (2023) employed rapamycin-regulated myeloid-derived suppressor cell exosomes (Rapa-Exo) in a corneal transplantation model. Their results demonstrated that Rapa-Exo conferred significantly superior therapeutic effects compared to unmodified exosomes, effectively prolonging graft survival, reducing corneal edema, and inhibiting pathological neovascularization. Mechanistically, this effect originates from the specific enrichment of miR-181d-5p in Rapa-Exo, which targets the transcription factor KLF6, thus inhibiting the production of downstream pro-inflammatory factors and the chemokine receptor CCR7. This discovery not only elucidates the mechanism of Rapa-Exo action but also identifies the miR-181d-5p/KLF6 signaling axis as a highly promising therapeutic target. Susmita Sahoo’s team (Sahoo et al., 2011) showed that CD34^+^ progenitor cell-derived exosomes accelerate limbal vascular network reconstruction, improving blood perfusion and nutrient supply, thereby enhancing graft survival.

Exosomes from diverse sources, including engineered variants, can synergistically improve corneal transplantation success by targeting both immune rejection inhibition and vascular reconstruction. These findings establish exosome-based strategies as a valuable approach in precision ophthalmology. While the majority of exosome-based strategies for transplant tolerance induction have focused on stem cell-derived vesicles, emerging evidence indicates that exosomes from non-stem cell sources can also exert potent immunomodulatory effects in corneal transplantation. As detailed in Section 5.2, exosomes derived from B16-F10 melanoma cells prolong allograft survival through JAK2-STAT-mediated activation of myeloid-derived suppressor cells, expanding the repertoire of potential therapeutic vehicles for combating graft rejection.

### 4.5 Relieve dry eye syndrome

DED constitutes a prevalent and multifactorial syndrome wherein instability of the tear film leads to injury of the ocular surface tissues. Clinically, it manifests as ocular discomfort, visual fatigue, and visual fluctuation. Inflammatory responses and osmotic stress are central to its pathogenesis (Craig et al., 2017). Stem cell-derived exosomes represent a promising therapeutic platform by virtue of their robust immunomodulatory and anti-inflammatory functions.

Multiple studies show that exosomes regulate innate immune and inflammatory signaling through various mechanisms. Wang et al. (2022) and Yu et al. (2020) reported that ADSC-Exos inhibit NLRP3 inflammasome activation, downregulating caspase-1, IL-1β, and IL-18, and alleviating ocular inflammation. Zhao et al. (2024) showed that BMSC-Exos deliver miR-21-5p to inhibit the TLR4/MyD88/NF-κB pathway, reducing pro-inflammatory cytokines (IL-17, IL-22) and increasing anti-inflammatory factors (IL-4, IL-10, TGF-β1), thereby mitigating DED. Wang et al. (2023b) identified ten regulatory miRNAs (*e.g.*, miR-125b-5p, let-7b-5p) in umbilical cord MSC-Exos that suppress the IRAK1/TAB2/NF-κB axis, alleviating ocular surface inflammation.

Guo et al. (2023) identified mechanisms regulating adaptive immune responses: MSC-Exos protect hCECs from high osmotic stress-induced damage and inhibit inflammatory factor expression *in vitro*. *In vivo*, MSC-Exos significantly reduced the severity of DED by suppressing dendritic cell (DC) maturation and the DC-mediated Th17 response.

Exosomes also show therapeutic value in pSS-associated autoimmune dry eye (Gliozzi et al., 2013). Li et al. (2022) demonstrated that MSC-Exos deliver miR-100-5p, promoting M2 macrophage polarization and increasing regulatory T Cells (Tregs), reshaping the immune microenvironment to enhance tear secretion and improve ocular surface health. The clinical relevance of these findings is supported by a prospective trial in which topical application of umbilical cord MSC-derived exosomes to 28 eyes of patients with refractory chronic graft-versus-host disease (cGVHD)-associated dry eye disease significantly relieved symptoms and reduced inflammation, mediated by miR-204 that reprograms M1 to M2 macrophages (Zhou et al., 2022). More recently, a randomized, triple-blind, placebo-controlled Phase I/II clinical trial evaluated the safety and efficacy of topical MSC-derived exosome eye drops in patients with pSS. In this trial, 8 patients received human Wharton’s jelly MSC-derived exosomes (10 µg/drop, twice daily for 2 weeks) in one eye and PBS in the contralateral eye. The treatment group demonstrated significant improvements in OSDI scores, tear secretion, tear film break-up time, and corneal fluorescein staining. Mechanistically, tear levels of epidermal growth factor (EGF) and thrombospondin-1 (THBS1) were significantly elevated, while pro-inflammatory IL-6 and MMP-9 were markedly reduced. No local or systemic adverse events were reported (Habibi et al., 2025).

In short, exosomes target key inflammatory signaling pathways (*e.g.*, NLRP3, TLR4/NF-κB) and regulate diverse immune cells (including DCs, Th17 cells, macrophages, and Tregs). By mitigating key pathological features of DED, these exosomes establish an emerging cell-free therapeutic paradigm with significant promise for treating refractory autoimmune cases.

### 4.6 Treatment of DK

DK is a common diabetic complication that affects vision but is often overlooked. A core mechanism is hyperglycemia-induced downregulation of miR-125a-5p, which triggers ER stress, inhibiting corneal epithelial cell migration, delaying wound healing, and compromising barrier function (Brown et al., 2019). Li et al. (2025) showed that BMSC-Exos compensate for the miR-125a-5p deficit under hyperglycemic conditions, inhibiting ER stress and ameliorating DK symptoms. This highlights a new class of exosome-based therapeutics targeting metabolic ocular diseases by modulating key miRNA pathways.

In addition, exosomes secreted by resident corneal cells help maintain homeostasis and facilitate repair. Verma et al. (2023) defined a precise role for normal limbal corneal epithelial cell exosomes (N-LEC-Exos) in modulating corneal limbal stem cells (LSCs) behavior. They found that N-LEC-Exos exert a triple effect on LSCs: promoting proliferation, enhancing migratory capacity (a key wound healing ability), and regulating the expression of phenotypic markers. This effect is environment-dependent. Under physiological conditions, N-LEC-Exos maintain the quiescence of LSCs through a dual mechanism: suppressing differentiation markers (*e.g.*, ALDH3A1) while concurrently upregulating stemness markers (*e.g.*, CD73, CD90, CD105). Conversely, under injury conditions, they promote a differentiation trend, indicating a role in guiding LSCs to participate in repair processes. This finding underscores the pivotal role of endogenous exosomes in the precise regulation of corneal limbal stem cell fate determination.

## 5 Exosomes derived from non-stem cell sources: functional roles and therapeutic potential

Corneal repair relies not only on exogenous stem cells but also on the intrinsic therapeutic potential of resident corneal cells and even microorganism-derived exosomes, offering a diverse array of cell-free therapeutic strategies.

### 5.1 Corneal cell-derived exosomes

Effective corneal repair is orchestrated by precisely coordinated interactions among its major constituent cell populations: hCECs, hCFs, and human corneal endothelial cells (hCEnCs). Exosomes serve as key mediators in this intercellular crosstalk.

#### 5.1.1 Functional diversity

Studies indicate that the exosomes derived from three cells synergistically promote corneal repair by activating distinct signaling pathways. hCECs-Exos effectively promote cell migration and proliferation by activating the STAT3, p38α (MAPK), and β-catenin pathways, along with STAT3/HSP27/p38α/Akt and GSK-3β/β-catenin signaling axes (Desjardins et al., 2022; McKay et al., 2020). HCFs-Exos primarily activate the HSP27 and GSK-3β/β-catenin pathways, thereby promoting migration and exerting anti-apoptotic effects, but lack STAT3-activating factors. HCEnC-Exos activate HSP27 while concurrently inhibiting the activities of GSK-3β, STAT5, and β-catenin, collectively promoting cellular stress adaptation. High TSP-1 levels in hCECs-Exos may promote myofibroblast differentiation, suggesting that modulating TSP-1 delivery could mitigate scar formation.

#### 5.1.2 Molecular mechanisms

Han et al. (2017) identified key functional proteins (*e.g.*, TSP-2, LTBP-1, CCL2, MMP2/TIMP1/TIMP2, Col6a3) in corneal epithelial exosomes. These proteins enhance TGF-β/Smad signaling, activate the PI3K/Akt and MAPK pathways, and precisely regulate the ECM degradation/synthesis balance via integrin-mediated FAK/Paxillin signaling. This coordinated action facilitates epithelial-stromal crosstalk and collectively regulates repair and scar formation. Furthermore, exosomal protein composition dictates biological effects. Han et al. (2020) found that the exosomal proteome was significantly remodeled in corneal fibroblasts following knockout of the MMP14 catalytic domain (Δexon4), underscoring the role of exosomal MMP14 in regulating corneal angiogenesis.

#### 5.1.3 Regulation of stem cell fate

Ramos et al. (2022) and Parekh et al. (2022) revealed deeper regulatory functions of corneal cell-derived exosomes. These exosomes regulate limbal stem cell homeostasis, differentiation, and proliferation by delivering specific miRNAs: miR-9-5p (which regulates HES-1 expression) and miR-196b-5p/miR-497-5p (which are enriched in pathways governing the cell cycle, adhesion junctions, and p53 signaling). Crucially, the exosome–miR-9-5p–HES-1 axis plays an important part in corneal wound repair. By delivering miR-9-5p, exosomes downregulate HES-1 expression, which relieves its suppression of cell differentiation. This process drives the transition of cells from a primitive progenitor state to a terminally differentiated corneal epithelial phenotype, thereby facilitating proper epithelial regeneration and maintaining ocular surface homeostasis. Notably, miR-196b-5p enhances p53 activity by targeting MDM2, thereby inhibiting endothelial cell proliferation.

### 5.2 Exosomes with immunomodulatory functions from diverse cellular sources

Notably, certain exosomes derived from non-stem mammalian cells exhibit unique immunoregulatory functions.

#### 5.2.1 B16 melanoma-derived exosomes

Exosomes derived from melanoma cells (B16-F10) represent a typical example. In a murine model of allogeneic corneal transplantation, subconjunctival injection of B16-Exos significantly prolonged graft survival, achieving a 70% survival rate at 42 days compared to only 20% in the PBS-treated control group. Proteomic analysis identified JAK2 as a central upregulated node in B16-Exos, which upon delivery to recipient myeloid-derived suppressor cells (MDSCs) activated the JAK-STAT signaling pathway, enhancing MDSC proliferation and immunosuppressive function. Concurrently, B16-Exo treatment markedly inhibited CD4^+^ T Cell proliferation, reduced corneal neovascularization by approximately 65%, and suppressed CD45^+^ immune cell infiltration, restoring corneal transparency to levels comparable to isogenic controls. Furthermore, these exosomes can inhibit tubule formation *in vitro*, an anti-angiogenic effect that further contributes to the prevention of graft rejection (Yang et al., 2025). This study establishes B16-Exos as a potent, naturally derived immunomodulatory agent and identifies the JAK2-STAT axis as a promising target for promoting transplant tolerance.

#### 5.2.2 Dendritic cell-derived exosomes in fungal keratitis

In infectious corneal diseases, exosomes from different sources exert distinct immunomodulatory effects. In a fungal keratitis model, thymic stromal lymphopoietin (TSLP)-treated DC exosomes deliver miR-21 to alleviate the suppression of Smad7. This enhances RORγt/IL-17 expression, promotes Th17 cell differentiation and inflammatory responses, and concurrently inhibits Foxp3/IL-10 expression and Treg function, ultimately exacerbating immunopathological damage (Ji et al., 2021). In contrast, during *P. aeruginosa* infection, corneal epithelial-derived exosomes recruit neutrophils via their chemotactic activity, promoting tissue recovery (Ramachandran et al., 2024).

#### 5.2.3 Human amniotic epithelial cell-derived exosomes

The therapeutic efficacy of exosomes derived from non-stem-cell sources, such as human amniotic epithelial cells (hAEC-Exos), has been rigorously validated. In dry eye models, hAEC-Exos confer multifaceted protection by enhancing epithelial proliferation and migration, suppressing inflammatory cytokines, and improving clinical parameters such as scores from corneal staining, tear secretion, and goblet cell density. Quantitatively, hAEC-Exos significantly promoted corneal epithelial cell proliferation (optical density increased from 0.95 to 1.24) and migration (wound closure rate elevated from 18.67% to 44.52%) within 48 h. Concurrently, they markedly downregulated key inflammatory cytokines: IL-1α, IL-1β, IL-6, TNF-α, and CCL2. Mechanistic investigations via proteomics and functional analyses revealed that these benefits are mediated through the activation of critical signaling pathways. HAEC-Exos treatment activated focal adhesion (indicated by enhanced FAK phosphorylation at Tyr397), PI3K-Akt signaling, ECM-receptor interaction, integrin signaling, and Wnt signaling.

In corneal alkaline burn models, the therapeutic effects of hAEC-Exos are attributed to their rich cargo of miRNAs (*e.g.*, miR-21-5p, miR-221-3p) and proteins (*e.g.*, TGFBI, FN1, TIMP2, ITGB1), which act synergistically to modulate ECM remodeling, activate focal adhesion, and inhibit the TGF-β pathway. Integrated multi-omics and experimental validation confirmed the direct transfer of hAEC-Exos cargo proteins (*e.g.*, FN1, TGFBI, TIMP2, CD44, COLIII) to corneal cells. This transfer enhanced ECM deposition and activated focal adhesion signaling in both *in vitro* and *in vivo* settings. At the molecular level, hAEC-Exos treatment upregulated pivotal focal adhesion components—including talin-1, paxillin, and tensin2—in corneal stromal cells, and induced FAK autophosphorylation (p-FAK Tyr397) in corneal epithelial cells. This coordinated signaling promotion facilitated epithelial regeneration, collagen reorganization, and scar attenuation (Yi et al., 2024; Hu et al., 2022).

However, exosome function is highly origin-dependent. Myofibroblast-derived exosomes overexpress CXCL1, CXCL6, CXCL12, MMP1, and MMP2, impairing epithelial migration, proliferation, and growth (Yeung et al., 2022). This finding underscores the critical importance of thorough functional validation prior to selecting an exosome source for therapeutic applications.

### 5.3 Microbially-derived extracellular vesicles: emerging therapeutic frontiers

Microbial extracellular vesicles (microbial EVs)—more precisely termed bacterial extracellular vesicles (BEVs), though often referred to as exosomes in the literature cited—are garnering increasing attention. For instance, vesicles derived from *Lactobacillus HY7302* demonstrated significant therapeutic potential in an experimental conjunctivitis model, reducing the expression of NFAT5 and NF-κB1 by 33% and 36%, respectively, and downregulating inflammatory mediators (for instance, IL-20, IL-8, IL-6, and IL-1β). These non-toxic microbial EVs are internalized by conjunctival cells via the “gut-eye axis,” mediating anti-inflammatory effects (Lee K. et al., 2024). This inter-tissue communication was functionally validated in a transwell co-culture system, where *HY7302* EVs crossed the intestinal epithelial barrier to suppress *benzalkonium chloride* (BAC)-induced inflammation in conjunctival cells, and is further supported by *in vivo* findings that oral *HY7302* administration ameliorated corneal damage in a dry eye mouse model while enriching Bifidobacterium pseudolongum in the gut microbiome.

Conversely, EVs from pathogens offer insights for therapeutic development. Tear exosomes isolated from a clinical cohort of 59 herpes simplex keratitis patients were found to carry the viral immediate-early protein ICP0, and their uptake by corneal epithelial cells led to the accumulation of ICP0 and ICP4 in recipient cells, identifying exosomes as both a latent reservoir and a cell-to-cell transmission vector for HSV-1 recurrence (Huang et al., 2023). Similarly, proteomic analysis of *Acanthamoeba* exosomes revealed novel virulence factors (*e.g.*, IUNH, proteasome subunits), providing a rationale for anti-amoebic drugs such as exosome secretion inhibitors (Lin et al., 2019).

Exosomes from commensal or beneficial microorganisms also show therapeutic potential. Salivary exosomes (SE) upregulate thrombospondin-1 (TSP-1) and cleaved vinculin (a wound healing marker) while downregulating fibrosis-related EDA-Fn in diabetic corneal models, suggesting they promote tissue healing via a vinculin-processing mechanism (Escandon et al., 2022). In a murine model of corneal epithelial debridement, topical administration of SEs (10 μg/eye, twice daily) significantly accelerated corneal wound closure within 24 h compared to PBS controls. *In vitro*, SEs enhanced the migration and proliferation of both HCECs and human limbal epithelial cells (HLECs) in a dose-dependent manner, while also improving mitochondrial oxidative phosphorylation capacity. Mechanistically, SE treatment transiently upregulated integrin α6, integrin β4, TSP-1, and TGF-β1 at 24 h post-wounding, with expression levels returning to baseline by 72 hours—a dynamic pattern that promotes initial wound repair while preventing excessive fibrosis (Liang et al., 2025). Notably, in primary corneal stromal cells from diabetic donors, these exosomes reduced fibrotic EDA-Fn expression and increased cleaved vimentin, indicating their capacity to correct aberrant wound healing phenotypes in patient-derived tissue and supporting their development as a non-invasive therapeutic for DK and other ocular surface disorders including dry eye disease.

## 6 Engineering modification and delivery system innovation of exosomes

**FIGURE 1:**
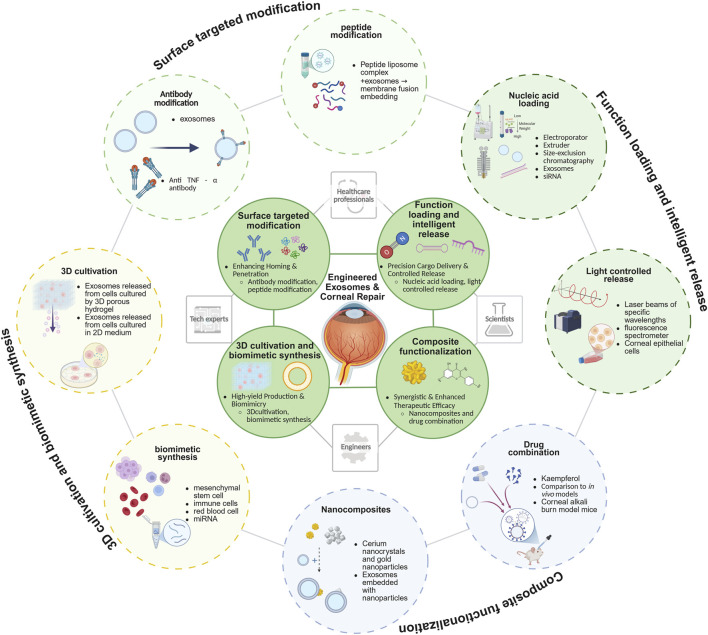
Schematic illustration of engineered exosomes as versatile platforms for targeted corneal therapy. This figure summarizes the multifaceted engineering strategies employed to improve the effectiveness of exosomes in treating corneal disorders. At the center, a human eye highlights the cornea as the primary target. These strategies are organized into two concentric rings: the inner ring outlines four core engineering objectives, while the outer ring details eight specific technical approaches that interconnect to achieve these goals. (Created in BioRender. hongkai, G (2026) https://BioRender.com/d5qurma).

Exosomes are emerging as ideal nanocarriers for next-generation drug delivery due to their biocompatibility, low immunogenicity, and innate ability to cross biological barriers. Gene editing and chemical modification techniques enhance exosome targeting precision, drug-loading capacity, and treatment efficacy while reducing off-target effects (Massoumi et al., 2023; Liang et al., 2021). Figure 1 provides a schematic overview of key engineering strategies for exosome functionalization discussed in this review.

### 6.1 Targeted modification strategies

**FIGURE 2:**
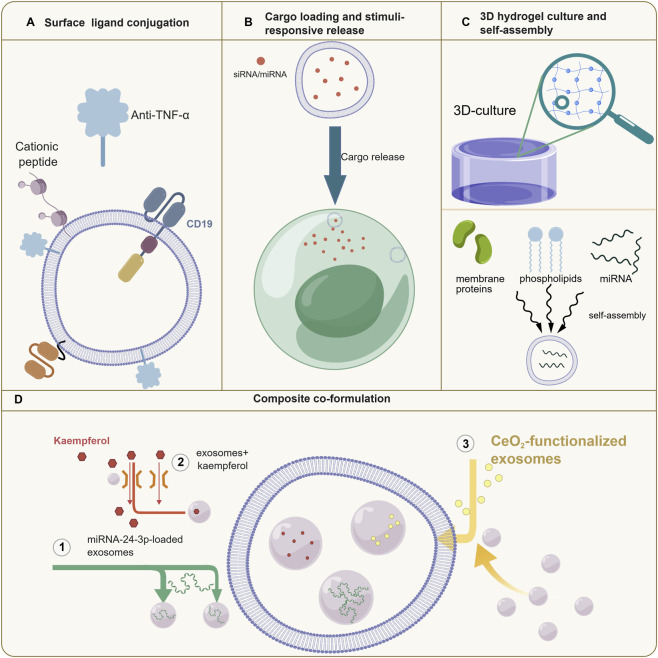
Representative experimental examples of exosome engineering strategies reported in the literature. While not exhaustive, these examples highlight key advances in **(A)** surface modification for targeted delivery, **(B)** functional cargo loading and stimuli-responsive release, **(C)** three-dimensional culture and biomimetic synthesis platforms, and **(D)** composite functionalization with small molecules, nanoparticles, or therapeutic RNAs. (Created with biogdp.com).

The part A of Figure 2 (Jiang et al., 2025) illustrates the principal surface engineering approaches for exosome targeting. Researchers have developed multiple engineering approaches including surface modifications and ligand conjugation to improve exosome targeting precision. Yu *et al.* created an aT-Exo complex by conjugating anti-TNF-α antibodies to ADSC-derived exosomes, achieving enhanced accumulation at corneal injury sites with improved inflammation resolution and tissue regeneration; delivery efficiency was further augmented using a polyvinyl alcohol microneedle system for deeper tissue penetration (Yu et al., 2024). Similarly, Millán Cotto *et al.* functionalized exosome surfaces with arginine-rich cationic peptides to address corneal and posterior ocular delivery barriers. The engineered exosomes doubled corneal diffusion rates, increased retinal photoreceptor uptake 10-fold, and improved eGFP mRNA transfection efficiency by 200%, while minimizing nonspecific diffusion in healthy lenses and preserving tissue selectivity and biocompatibility (Cotto et al., 2024). These surface engineering approaches, along with the cargo loading and composite functionalization strategies schematically outlined in Figure 1, collectively expand the therapeutic repertoire of exosome-based therapies.

### 6.2 Functional cargo loading and intelligent delivery systems

Figure 2B summarizes key strategies for therapeutic cargo loading and stimuli-responsive release from engineered exosomes. Loading exosomes with functional molecules significantly enhances their therapeutic potential. Xie *et al.* developed a hybrid extracellular vesicle system by fusing siRNA-loaded liposomes with corneal epithelial cell exosomes, achieving effective intracellular delivery and *NFKBIZ* silencing (Xie et al., 2024). The delivered siRNA suppressed inflammatory factors and remodeled the immune microenvironment, effectively alleviating dry eye symptoms. Liu *et al.* engineered MSC-derived exosomes loaded with a miR-29b-3p agomir (Exos-29b-ago) to leverage its role in regulating autophagy and fibrosis pathways. In a corneal injury model, Exos-29b-ago demonstrated potent anti-inflammatory effects through dual regulation of the PI3K/AKT/mTOR inhibition and mTOR/NF-κB/IL-1β pathway activation, achieving significantly stronger effects than antagonist and control groups (Pu et al., 2022; Liu et al., 2024).

Regarding the intelligent responsive delivery systems, Duan *et al.* developed photoresponsive polymer vesicles for corneal repair. The system employs a novel N-nitrosamine NO donor (oNBN/pNBN/BN) that effectively resolves NO stability issues and prevents premature leakage. This innovative system enables light-triggered NO release that promotes corneal epithelial migration and proliferation while accelerating wound healing without cytotoxicity, supporting safe and effective tissue repair (Duan et al., 2020).

### 6.3 Three-dimensional culture and biomimetic synthesis strategies

Figure 2C depicts two advanced production platforms: 3D hydrogel culture for enhanced yield and self-assembly of synthetic exosomes. To overcome the bottlenecks of low yield and limited functionality associated with traditional two-dimensional (2D) culture-derived exosomes, Xu and Jailian developed three-dimensional (3D) cultured MSC exosomes (3D-Exos) using a gelatin methacryloyl hydrogel system. Compared to 2D-exosomes, 3D-Exos significantly upregulated dry-eye markers (P63/PAX6) and corneal stem cell functional proteins, downregulated myofibroblast markers, and promoted macrophage polarization towards the M2 phenotype. Consequently, 3D-Exos exhibited a superior overall capacity to reduce scarring, alleviate inflammation, and accelerate corneal wound healing (Xu et al., 2025; Jalilian et al., 2022).

Furthermore, biomimetic synthesis strategies provide novel avenues for exosome applications. Unlike naturally derived exosomes, these fully synthetic vesicles are assembled from defined lipid and protein components using bottom-up engineering approaches, enabling precise control over composition, size, and surface functionalization. This design flexibility circumvents the compositional heterogeneity inherent to biological exosome preparations and facilitates standardized, scalable manufacturing—critical prerequisites for clinical translation. By incorporating specific lipids, membrane proteins, and therapeutic cargo molecules during the self-assembly process, synthetic exosomes can be tailored for targeted drug delivery to corneal tissues. Staufer *et al.* developed top-down synthesized, fully synthetic exosome—like vesicles capable of efficiently delivering functional molecules like miR-132. By inhibiting *TIMP3* and activating the integrin signaling pathway, exosomes effectively enhance cell migration and proliferation (Staufer et al., 2021).

### 6.4 Composite functionalization and exosome efficacy enhancement

Beyond the cargo-loading strategies discussed in Section 6.2, where therapeutic molecules are incorporated into individual exosomes, composite functionalization involves combining intact exosomes with other active ingredients or materials to achieve synergistic therapeutic effects. Figure 2D outlines composite co-formulation strategies in which exosomes are combined with small molecules, nanoparticles, or therapeutic RNAs for synergistic therapeutic effects. Combining exosomes with other active ingredients or materials can synergistically enhance their therapeutic efficacy. Liu *et al.* combined platelet exosomes with kaempferol, effectively inhibiting corneal neovascularization and inflammatory responses (Liu et al., 2025). Tian *et al.* synthesized cerium nanocrystal-functionalized exosomes via an *in situ* growth method. This complex exhibits potent reactive oxygen species (ROS) scavenging capacity and storage stability without requiring a cell nucleus. It significantly enhances the corneal re-epithelialization process and demonstrates excellent efficacy in treating DED (Tian et al., 2023). Sun *et al.* combined the anti-inflammatory and proliferative properties of ADSC-Exos with the migration-promoting function of miR-24-3p to create Exos-miR-24-3p. These engineered exosomes not only accelerated corneal epithelial wound closure but also restored tissue structure, cellular maturity, and the expression of cytokeratin CK3 and tight junction protein ZO-1 to normal physiological levels (Sun et al., 2023; Yu et al., 2017; Kang et al., 2021).

Ma *et al.* developed an mExo@AA system. This system minimizes excessive gold nanoparticle deposition on MSC-Exosome membranes via *in situ* ascorbic acid reduction, significantly enhancing their biological efficacy. This system reduced corneal ROS levels by 80%, increased the M2 macrophage proportion to 56.72%, and enhanced corneal epithelial cell migration, achieving a wound closure rate of 70.9%. These results demonstrate its unique therapeutic value for dry eye disease (Ma et al., 2023).

Finally, exosomes have emerged as a highly customizable drug delivery platform through strategies including genetic engineering, chemical modification, 3D culture, and intelligent material composites. These engineering strategies not only significantly enhance exosome targeting and therapeutic efficiency but also broaden the prospects for future clinical translation. Together with the targeted modification and cargo loading strategies depicted in Figure 1, these emerging production and functionalization platforms are progressively addressing the key translational bottlenecks of exosome-based therapies.

### 6.5 Barriers to clinical translation: delivery hurdles, toxicity, and regulatory considerations

Despite remarkable progress in exosome engineering, several fundamental challenges must be addressed before these nanocarriers can achieve widespread clinical application.

#### 6.5.1 Intrinsic delivery barriers

The therapeutic efficacy of systemically administered exosomes is significantly limited by rapid hepatic clearance. Approximately 80% of intravenously injected exosomes are sequestered by the liver within hours, primarily via Kupffer cell and hepatocyte scavenger receptors (Schank et al., 2026; Zhang and Fang, 2026). Additional barriers include protein corona-mediated masking of targeting ligands, limited stromal penetration in fibrotic corneas, and rapid renal clearance of sub-50 nm exosomes. Strategies such as CD47 functionalization, PEGylation, and targeted peptide conjugation represent direct responses to these hurdles (Zhang and Fang, 2026).

For topical ocular delivery, additional anatomical and physiological barriers unique to the corneal surface present formidable challenges. The multilayered structure of the corneal epithelium, with its tight intercellular junctions, restricts the penetration of nanoparticles larger than 50 nm. Moreover, dynamic factors—including a tear turnover rate that clears approximately 60% of an applied dose within 2 min, reflex blinking, and nasolacrimal drainage—collectively limit drug retention and bioavailability, with less than 5% of a topically administered dose typically reaching intraocular tissues (Xie and Jie, 2025). These barriers are particularly problematic for exosome-based therapies, as the lipid bilayer, while protective of cargo, does not inherently confer the ability to traverse the intact corneal epithelium.

Several engineering strategies have been developed to address these ocular delivery hurdles. Cationic motif-modified exosomes, engineered by anchoring arginine-rich peptides to the exosomal surface via PEG 2000 lipid insertion, exploit electrostatic interactions with the anionic glycosaminoglycans of the corneal epithelium and vitreous humor. This modification has been shown to double corneal diffusion rates and increase retinal photoreceptor uptake by 10-fold in *ex vivo* models, while minimizing nonspecific diffusion in healthy lenses (Gamez et al., 2025). For corneal injuries involving epithelial defects, where the barrier is partially compromised, microneedle-based delivery systems offer an alternative route. Yu *et al.* developed polyvinyl alcohol microneedles loaded with anti-TNF-α-engineered ADSC-derived exosomes, achieving enhanced accumulation at corneal injury sites with improved inflammation resolution and tissue regeneration compared to topical administration alone (Xie and Jie, 2025). Additionally, 3D bioreactor culture of MSCs has been shown to increase EV yield by up to 100-fold compared to conventional 2D culture, thereby addressing the scalability bottleneck that must be solved in tandem with delivery efficiency to enable clinical translation (Massoumi et al., 2026).

#### 6.5.2 Toxicity and immunogenicity

While MSC- and DC-derived exosomes demonstrate favorable safety profiles in early trials, tumor-derived exosomes carry oncogenic risks (VEGF, MMPs, PD-L1, miR-21), and plant-derived exosomes may trigger immunogenicity via species-specific lipids (Zhang and Fang, 2026). Engineering processes introduce additional risks: residual PEG, electroporation-induced membrane damage, and transfection reagent contamination all require rigorous quality control. Critically, long-term toxicity data—including chronic, reproductive, and carcinogenicity assessments—remain absent (Schank et al., 2026).

#### 6.5.3 Regulatory and standardization hurdles

Without dedicated regulatory frameworks, exosome-based products must navigate Good Manufacturing Practice (GMP) standards designed for cell therapies. The FDA regulates EVs as biologics/drugs under the PHS Act, while the EMA classifies them on a case-by-case basis, generally as biological medicinal products or ATMPs if they contain recombinant nucleic acids (Verma and Arora, 2025). In Asia, regulatory approaches diverge: Japan’s Pharmaceuticals and Medical Devices (PMD) Act permits conditional, time-limited approvals, whereas South Korea and Taiwan have issued EV-specific guidelines. A recent analysis of the WHO ICTRP database reveals that 61% of registered EV-based trials originate from China, followed by the U.S. (17%) and Japan (7%), underscoring the global but uneven distribution of translational activity. Key pain points include undefined potency assays, batch-to-batch cargo variability, and unstandardized quality attributes. The MISEV2023 five-component framework—requiring reporting of source, isolation parameters, quantitative metrics, positive markers (≥2 transmembrane + 1 cytosolic protein), and negative markers (≥1 non-EV contaminant)—offers foundational guidance, but harmonization across settings remains elusive (Choi et al., 2026).

#### 6.5.4 Manufacturing and scalability challenges

Large-scale GMP-compliant production represents a critical bottleneck. Stirred-tank bioreactors combined with TFF enable closed, continuous concentration with >95% recovery, while hollow-fiber systems can increase exosome yield up to 100-fold compared to static 2D culture (Kimiz-Gebologlu and Oncel, 2025; Schank et al., 2026). A scalable workflow integrating TFF with SEC achieves particle-to-protein ratios of ∼1 × 10^9^ particles/μg protein, substantially reducing albumin contamination (Lundy et al., 2026; Kimiz-Gebologlu and Oncel, 2025). Emerging technologies such as EXODUS—a negative-pressure oscillation system—accomplish high-purity exosome isolation within 5 min without compromising vesicle integrity. For engineered exosomes, optimized electroporation (400 V, 150 μF, 1 ms) or sonication followed by 37 °C membrane recovery maximizes loading efficiency while minimizing damage. Critical quality attributes for batch release include particle size distribution, identity markers (CD9/CD63/CD81), sterility, endotoxin (<5 EU/kg/h), and functional potency assays.

#### 6.5.5 Therapeutic challenges and emerging solutions

Beyond manufacturing and regulatory hurdles, several therapeutic challenges demand attention: (1) pronounced functional heterogeneity across exosome sources, with core mechanisms incompletely understood; (2) complex workflows for engineered exosome production, hindering scalable GMP manufacturing; (3) suboptimal corneal targeting efficiency, with risks of inadequate stromal penetration or off-target distribution; (4) potential carriage of pro-inflammatory or pathogenic cargo (*e.g.*, HSV-1 genes) under specific disease conditions; and (5) the need for comprehensive long-term safety evaluation. Promising optimization strategies include: standardized production incorporating physical stimulation to enhance yield and functional consistency; intelligent engineered exosomes with MMP-cleavable linkers for inflammation-triggered drug release; and novel delivery systems such as dissolvable microneedle patches to bypass the corneal barrier and improve topical bioavailability.

## 7 Conclusion

Exosomes represent a versatile class of natural nanocarriers with substantial diagnostic and therapeutic potential for corneal diseases. Their cell-specific cargo reflects physiological and pathological states, enabling early disease detection, while their intrinsic capacity to mediate intercellular communication supports tissue repair, inflammation resolution, and targeted drug delivery. As detailed throughout this review, therapeutic efficacy has been demonstrated across diverse sources—from stem and immune cells to commensal microorganisms—operating through pathways that include HIPK2/JNK, TGF-β/Smad, NF-κB, and the gut-eye axis. Engineered exosomes further extend this potential through surface modification, cargo loading, and intelligent delivery systems that enhance corneal retention and therapeutic precision.

However, clinical translation remains constrained by challenges that span the entire development pipeline. Isolation methodologies inevitably involve trade-offs between purity, yield, and scalability, while the inherent heterogeneity of EV populations confounds batch-to-batch reproducibility and mechanistic interpretation. The dual role of exosomes in both healing and pathogenesis demands rigorous source validation. Furthermore, the absence of standardized characterization frameworks and harmonized regulatory pathways impedes large-scale GMP manufacturing and multinational clinical development.

Addressing these barriers will require concerted advances in three priority areas: (i) the development and adoption of standardized, high-purity isolation workflows guided by MISEV2023 recommendations; (ii) rigorous elucidation of the molecular mechanisms governing exosome-mediated corneal repair, supported by single-vesicle analytics and machine learning; and (iii) refinement of engineering strategies to enhance targeting precision, cargo delivery efficiency, and long-term safety. Through interdisciplinary innovation, exosomes hold transformative potential as powerful biological tools for the detection and therapy of corneal diseases.

## 8 Discussion

While exosome research has advanced significantly, investigations into their role in corneal contexts require further depth. The diagnostic value of exosomes derives from their content of diverse biomolecules (proteins, lipids, DNA and RNA, *etc.*), which exhibit specific alterations under physiological or pathological conditions. These characteristics position exosomes as promising tools for early disease warning and diagnosis. The isolation method profoundly influences which cargo is detected: ultracentrifugation co-sediments protein aggregates that may obscure low-abundance biomarkers (Choi et al., 2026; Lundy et al., 2026), while size-exclusion chromatography preserves vesicle integrity but dilutes the sample, and immunoaffinity capture selectively enriches only subpopulations bearing the targeted marker. Thus, the choice of isolation strategy directly determines the diagnostic sensitivity and specificity achievable in downstream analyses.

Therapeutically, exosomes coordinate corneal repair via mechanisms dictated by their cellular origin. Stem cell-derived exosomes promote tissue regeneration and mitigate fibrosis primarily through miRNA-mediated regulation of key pathways, such as HIPK2/JNK and TGF-β/Smad. Immune cell-derived exosomes modulate inflammatory responses by targeting NF-κB signaling and influencing macrophage polarization. Corneal tissue-derived exosomes, in contrast, facilitate epithelial-stromal crosstalk and repair via pathways that include HSP27/p38 MAPK. Furthermore, microbial and viral components can exploit EVs for pathogenesis, underscoring the dual role of these vesicles in both therapy and disease progression. This duality mirrors a broader theme—exosomes are inherently neutral vehicles whose functional outcome depends entirely on their source and cargo. Crucially, engineered exosomes not only enhance these inherent therapeutic activities but also enable precise drug delivery, offering advantages unattainable by conventional therapeutic approaches.

Nevertheless, the isolation and purification of exosomes remain a critical bottleneck. Existing methodologies present inherent limitations, failing to achieve an optimal balance between efficiency and high specificity: ultracentrifugation is widely used but time-consuming and yields limited purity; ultrafiltration is rapid yet may compromise exosome integrity; precipitation is cost-effective but suffers from low yield; immunoaffinity capture offers high specificity but is constrained by antibody/bead availability and low throughput and size-exclusion chromatography is gentle and yields high purity, yet involves substantial equipment costs. Collectively, current techniques necessitate trade-offs between purity, yield, operational complexity, and cost, underscoring the urgent need for standardized, high-throughput, and cost-effective isolation strategies.

To address these challenges, researchers are pursuing advances through methodological integration and parameter optimization. A core strategy involves establishing a workflow from crude isolation to refined purification. For instance, combining low-cost PEG precipitation for rapid concentration with high-resolution size-exclusion chromatography for fine purification can significantly enhance final product purity while maintaining high yield and biological activity. Fine-tuning established methods is equally critical. The common practice of adjusting ultracentrifugation time by K-factor is invalid for fixed-angle rotors; instead, a cut-off size approach based on complete sedimentation of a desired vesicle diameter should be employed. Furthermore, adopting unified, optimized protocols as standard operating procedures and exploring automated microfluidic technologies represent fundamental directions for achieving high-throughput, reproducible separations. Emerging techniques such as asymmetric flow field-flow fractionation enable nanometer-scale resolution without shear stress, while tangential flow filtration coupled with size-exclusion chromatography achieves substantially improved purity metrics that align with GMP-compliant manufacturing requirements (Lundy et al., 2026).

In the therapeutic domain, despite their significant promise for corneal diseases (*e.g.*, promoting regeneration, anti-inflammation, targeted drug delivery), exosomes face substantial challenges: (1) Functional heterogeneity across different exosome sources is pronounced, and their core mechanisms of action are incompletely understood, demanding further validation of safety and reproducibility for clinical application; (2) The preparation of engineered exosomes (involving techniques like genetic editing and chemical modification) entails complex processes, posing difficulties for scalable manufacturing and raising concerns about stability; (3) The corneal targeting efficiency of exosomes as drug carriers requires improvement, with risks of inadequate local penetration or off-target distribution; (4) Significant caution is warranted regarding the potential for exosomes to carry pro-inflammatory factors or pathogen components (*e.g*., HSV-1 viral genes) under specific pathological conditions (such as fungal keratitis or viral infections), posing risks of exacerbated inflammation or infection transmission; (5) Finally, while exosomes exhibit low immunogenicity, the systemic implications and long-term safety profile of their use necessitate comprehensive evaluation. These five challenges correspond directly to the translational barriers facing the field: the delivery hurdles of rapid hepatic clearance and protein corona masking, the regulatory and manufacturing bottlenecks of GMP compliance, and the toxicity concerns surrounding source-dependent oncogenic or immunogenic risks. Feasible optimization strategies include establishing standardized production protocols utilizing physical stimulation to enhance yield and functional consistency, developing intelligent engineered exosomes with MMP-cleavable peptide linkages for inflammation-triggered drug release, and employing novel delivery systems such as dissolvable microneedle patches to bypass the corneal barrier and improve topical bioavailability. These strategies align with emerging single-vesicle analytics and machine learning-assisted quality control approaches, which together offer a path toward resolving heterogeneity and achieving clinical-grade consistency.

It is imperative to note that the current literature often conflates the distinct concepts of “exosomes” and “(extracellular) vesicles,” which risks serious misinterpretation in subsequent research. Future investigations should prioritize: developing highly precise isolation technologies, elucidating the core molecular mechanisms underpinning exosome function, and refining engineering strategies to enhance therapeutic targeting and safety. Through interdisciplinary innovation, exosomes hold transformative potential as breakthrough biological tools in the detection and therapy of corneal diseases.
